# The Adaptation Model Offers a Challenge for the Predictive Coding Account of Mismatch Negativity

**DOI:** 10.3389/fnhum.2021.721574

**Published:** 2021-11-19

**Authors:** Patrick J. C. May

**Affiliations:** Department of Psychology, Lancaster University, Lancaster, United Kingdom

**Keywords:** adaptation, auditory cortex, mismatch negativity, MMN, N1, predictive coding, stimulus omission

## Abstract

An unpredictable stimulus elicits a stronger event-related response than a high-probability stimulus. This differential in response magnitude is termed the mismatch negativity (MMN). Over the past decade, it has become increasingly popular to explain the MMN terms of predictive coding, a proposed general principle for the way the brain realizes Bayesian inference when it interprets sensory information. This perspective article is a reminder that the issue of MMN generation is far from settled, and that an alternative model in terms of adaptation continues to lurk in the wings. The adaptation model has been discounted because of the unrealistic and simplistic fashion in which it tends to be set up. Here, simulations of auditory cortex incorporating a modern version of the adaptation model are presented. These show that locally operating short-term synaptic depression accounts both for adaptation due to stimulus repetition and for MMN responses. This happens even in cases where adaptation has been ruled out as an explanation of the MMN (e.g., in the stimulus omission paradigm and the multi-standard control paradigm). Simulation models that would demonstrate the viability of predictive coding in a similarly multifaceted way are currently missing from the literature, and the reason for this is discussed in light of the current results.

## Introduction

Change detection in the brain is studied by using the oddball paradigm where sporadically presented deviant stimuli are mixed in among often-repeating standard stimuli. The brain tends to respond weakly to standards and vigorously to deviants. In event-related potential (ERP) and field (ERF) measurements, the mismatch negativity (MMN) is defined as the difference in the respective responses elicited by deviants and standards. Despite the simplicity of this technical definition, there is nothing simple nor self-evident about the MMN. This is because it reflects two fundamental aspects of brain function: the flair for representing the world in terms of patterns, and the ability to pick out pattern-breaking events that carry the promise of salience. Butler (1968) originally described the differential between the standard and deviant N1 (“V”) responses and explained in terms of neuronal habituation which selectively suppresses those neurons tuned to the standard. Näätänen et al. (1978) named this differential the MMN and suggested that it reflects the operation of sensory memory. Näätänen (1990, 1992) proposed a model of two-tier processing where the adherence of the stimulus to a repeating pattern is evaluated in a dedicated MMN generator, and where the suppression of the N1 happens in a separate generator which registers stimulus onsets. A rival explanation, the so-called adaptation model, is similar to Butler’s interpretation and suggests that suppressive effects within auditory cortex can account for the MMN and that the MMN is part of a modulated N1 response (May et al., 1999, 2015; Jääskeläinen et al., 2004; May and Tiitinen, 2010; Fishman, 2014). It is unclear what the physiological mechanisms of cortical adaptation/suppression are, but a likely candidate is short-term synaptic depression, STSD (Wehr and Zador, 2003, 2005). This has decay times up to several seconds, which coincides with the time constants of stimulus-specific adaptation (SSA) measured intracortically (Ulanovsky et al., 2003, 2004).

The MMN has edged its way toward mainstream neuroscience, helped along by its new-found role as a prime specimen of predictive coding (PC). This posits that perception is essentially an inference problem which the brain solves by constructing “generative models” to explain the causes of the sensory input (Rao and Ballard, 1999; Friston, 2005, 2010; Bastos et al., 2012). Such models sit at the top of the brain’s processing hierarchy and generate prediction signals that are passed down the hierarchy. At each level, these signals attempt to match the sensory signals making their way up the hierarchy. When this matching occurs, the successful prediction signal suppresses the sensory signal. If there is a mismatch between the two, the sensory signal remains unsuppressed and continues its upwards journey. Therefore, sensory responses inform the system that a prediction error has occurred and that the generative model needs updating. Perception is a process where error signals nudge generative models into forms which minimise the prediction error, thereby offering the best explanation of the sensory input. In this framework, the repetition suppression of the N1 response to the standard is due to a dampening of the sensory signal by a successful prediction signal (Auksztulewicz and Friston, 2016) and the MMN to the deviant is a prediction error signal (Garrido et al., 2009; Wacongne et al., 2012; Chennu et al., 2013; Lieder et al., 2013a,b; Rentzsch et al., 2015; Carbajal and Malmierca, 2018; Fong et al., 2020).

The rise of PC as an explanation of the MMN has been heralded by a number of studies which point to evidence in favour of PC and against the adaptation model (e.g., Wacongne et al., 2012; Lieder et al., 2013a; Fitzgerald and Todd, 2020). Here, we revisit this issue and consider the viability of PC obliquely: I present the modern version of the adaptation model and a variety of simulations which produce MMN responses, including some that might pose a challenge for PC.

## The Adaptation Model Comes in Vanilla and Chocolate

There are two varieties of adaptation model. Its most common form is also the traditional and most simplistic one. It builds on the premise that neurons that are repetitively stimulated become less responsive. The traditional model takes a unit-centric view by extrapolating this behaviour to event-related responses. The MMN is explained by the populations tuned to the standard being more adapted than those tuned to the deviant. The response to the stimulus is then a bottom-up process where the sensory signal drives the neural population to respond with a magnitude that depends on the adaptation level. Further, it is assumed that adaptation on both the unit and the population level depends on one aspect only: the time series of the specific stimulus to which the population is tuned. Thus, other stimuli used in the paradigm do not affect the responsiveness of the population. This traditional adaptation model is unconvincing (Fitzgerald and Todd, 2020): It can’t explain the mismatch response to stimulus omissions (Yabe et al., 1997, 1998), because the responses of the model require a sensory signal. Also, it fails to explain the MMN to unexpected stimulus repetitions (Wacongne et al., 2012) because stimulus repetition supposedly always leads to more adaptation and a weaker response.

The traditional adaptation model can be operationalised to produce predictions of evoked responses. For example, Lieder et al. (2013a) formulated the adaptation hypothesis as exponentially adapting and recovering frequency channels and found that the experimental data favoured a model based on PC. Moreover, this idea of isolated adapting frequency channels is the basis for the multi-standard control paradigm (Schröger and Wolff, 1996; Jacobsen and Schröger, 2001). Here, the oddball condition is complemented by a control condition where the standards are replaced by several different stimuli equiprobable with the deviant. Because the presentation rate of the deviant is identical across the two conditions, the level of adaptation, according to the traditional adaptation model, should also be identical. Therefore, if the response to the deviant is stronger in the oddball condition than in the multi-standards control condition, this is taken as unequivocal proof that adaptation cannot explain the MMN, and that the MMN must therefore reflect something more. The multi-standard control condition has produced plenty of evidence that apparently refutes the adaptation model (for a review, see May and Tiitinen, 2010). It has recently become popular in animal electrophysiology where it is used for demonstrating that mismatch responses cannot be explained in terms of stimulus-specific adaptation (e.g., Harms et al., 2014; Kurkela et al., 2018) and that PC is therefore a more likely explanation (e.g., Parras et al., 2017).

There is a modern version of the adaptation model which bears but passing resemblance to its traditional counterpart. The acorn for this was planted by May et al. (1999) who argued that the frequency MMN can be explained as a modulated N1 response being generated on tonotopic maps with post-stimulus inhibition. The study used a computational model of auditory cortex where individual microcolumns interact with each other through lateral connections. This departure from the traditional adaptation model yielded a prediction, verified in EEG measurements, that the peak latency of the response to the deviant should have a non-monotonic dependence on the standard-deviance separation. This idea of modeling the auditory cortex as a system of interacting units (rather than isolated channels) was further developed by May and Tiitinen (2010) in their treatise on the adaptation model. These authors noted that the results which initially might appear to falsify the adaptation model are in fact consistent with this model. For example, the activity associated with the response to the standard has a different spatial distribution than the activity underlying the MMN, (e.g., Rinne et al., 1999). Findings such as these have been used as evidence against the adaptation model as they appear to show that the generators of the MMN are separate from those of the N1 (for a review, see Näätänen et al., 2005). However, the adaptation model offers a simpler explanation in terms of variations in stimulus selectivity across cortical fields. For example, a field with broadly tuned neurons will respond similarly to the standard and deviant while, at the same time, a field with sharply tuned neurons will show stronger activation to the deviant. The spatial distribution of the responses elicited by the standard and deviant will therefore differ without implying the existence of a dedicated MMN generator (see sections 6.2 and 6.3 of May and Tiitinen, 2010). Further, one of the themes put forward by May and Tiitinen was that synaptic depression operating in the interconnected system of auditory cortex makes the system’s responses highly context-dependent. This dependence shows up as MMN responses of various kinds as well as stimulus selectivity on the single-unit level. Staying on this theme, May and Tiitinen (2013) introduced a computational model that structurally copies the gross anatomy of the auditory cortex and where the synapses are modulated by STSD. Simulations showed that this system performs temporal binding, with individual columns exhibiting combination sensitivity similar to that found in monkey auditory cortex (Rauschecker, 1997). This sensitivity was found to be caused by the combination of STSD and the serial core-belt-parabelt structure of auditory cortex. In further simulations (May et al., 2015), the model replicated single-unit forward masking and SSA (Ulanovsky et al., 2003, 2004) as well as forward facilitation (Brosch et al., 1999; Brosch and Schreiner, 2000). Further, the model reproduced repetition suppression of the N1 (Lü et al., 1992) as well as several types of MMN. These were the frequency MMN (Tiitinen et al., 1994), MMN to “abstract” sound features (Korzyukov et al., 2003), and the MMN to small changes in complex tone sequences (Näätänen et al., 1993), where the latter two types are classed as evidence for “primitive intelligence” of auditory cortex (Näätänen et al., 2001). The success of the model in being able to recreate such a wide variety of phenomena was found to be a consequence of STSD. Removing STSD also abolished SSA, masking, facilitation, combination sensitivity, N1 adaptation, and the MMN.

## The Adaptation Model in Action: Simulation Methods

**FIGURE 1:**
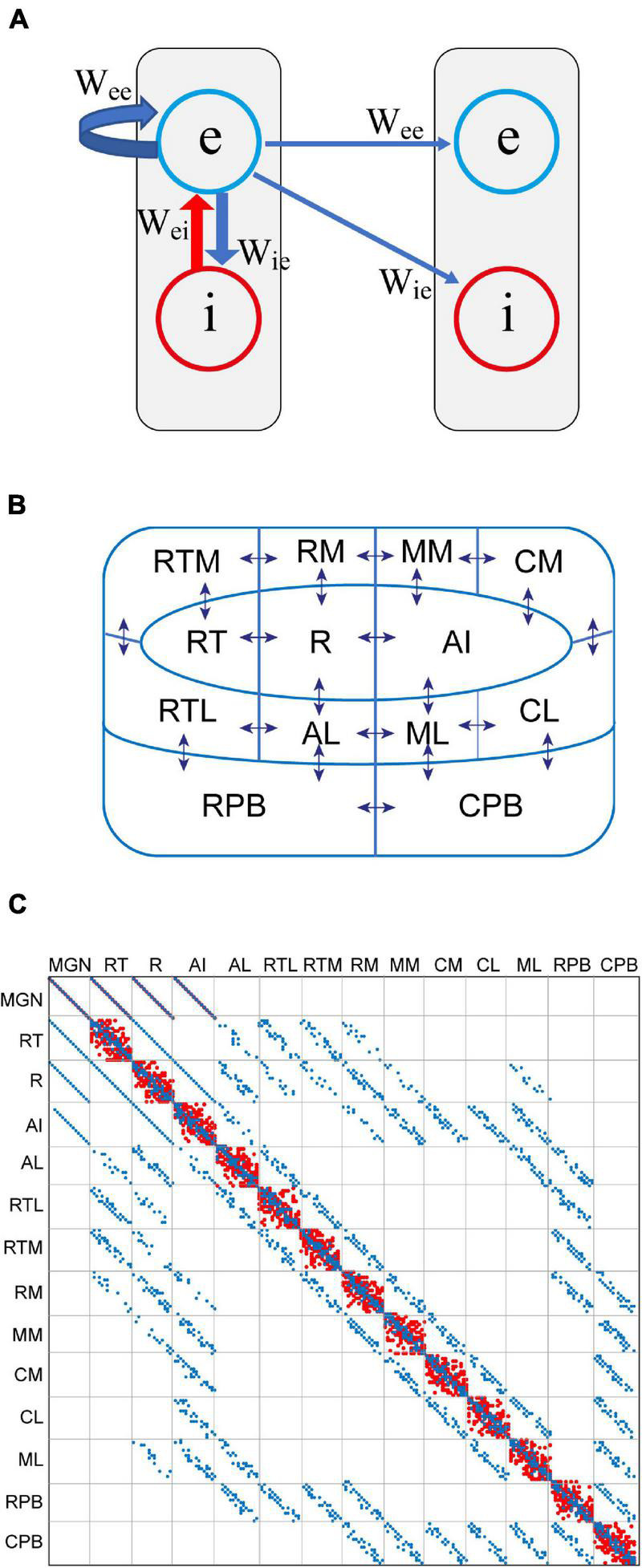
A computational model of auditory cortex as a modern version of the adaptation model. **(A)** The basic functional unit of the model is the cortical column. This comprises a lumped description of the excitatory (e) and inhibitory (i) neuron populations. The e-population connects back to itself via feedback connections described in the weight matrix **W**_ee_. It also excites the excitatory populations of other columns. Lateral inhibition occurs through the e-population driving the i-population of neighbouring columns. **(B)** There are 208 cortical columns organised into three core fields (R, RT, AI), eight belt fields (AL, RTL, RTM, RN, MM, CM, CL, ML), and two parabelt fields (RPB, CPB). Neighbouring fields are strongly interconnected, as indicated by the arrows. The connections from RPB to RTM and RM as well as those from CPB to RM, MM, and CM are not shown. Abbreviation key: A – anterior (except for AI, primary auditory cortex), R – rostral, C – caudal, M – medial, L – lateral, T – temporal, PB – parabelt. **(C)** The weight matrices **W**_ee_ (blue) and **W**_ie_ (red) are overlayed. **W**_ie_ mediates lateral inhibition within each field. Long-range connections are found in **W**_ee_ only. Feedforward connections are below the diagonal, and feedback connections are above it.

Original simulations of the modern version of the adaptation model were carried out to demonstrate that it reproduces those types of MMN which previously have been taken as evidence against the adaptation hypothesis. Importantly, these MMN responses, both empirically observed and simulated, might pose a challenge for the PC model as currently formulated. The model here is a modification of that of auditory cortex introduced in May and Tiitinen (2013) and May et al. (2015). It has a hierarchical structure with feedforward and feedback connections between cortical fields. However, the resemblance to PC stops here, there being no separate prediction and error units. Instead, as shown in Figure 1A, the dynamical unit of the model is a simplified description of the cortical column. Within each column, the excitatory and inhibitory neurons are treated as lumped populations described by mean-field state variables *u*(*t*) and *v*(*t*), respectively. These variables correspond to the membrane potential, and they are transformed into the mean firing rate through *g*(*x*) = 1[*x* –θ]tanh[2(*x* – θ)/3], where θ = 0.05 is the threshold for firing and 1[.] is the Heaviside step function.

As depicted in Figure 1B, there are 208 cortical columns arranged into three core fields, eight belt fields, and two parabelt fields, with 16 columns per field (see Hackett et al., 2014). In addition, there is a 16-unit field where the excitatory populations represent the medial geniculate nucleus (MGN) of the thalamus and the inhibitory populations represent the surrounding thalamic reticular nucleus (for details, see Hajizadeh et al., 2019). There are therefore a total of 224 units. Fields are connected topographically to each other according to the anatomical results of Hackett et al. (2014). The signal progresses along the feedforward connections by first entering the MGN which then targets the three core fields, and these project to the surrounding belt fields, which in turn are connected to the two parabelt fields. These forward connections are reciprocated by feedback connections. Anatomically neighbouring fields are strongly interconnected while obliquely situated fields have fewer interconnections. The rostral parabelt field is interconnected with the anterior belt fields, and the caudal parabelt field connects with the posterior belt fields.

As illustrated in Figure 1C, the connectivity between the fields is expressed in the way the populations of excitatory neurons are connected to each other according to the 224 × 224 weight matrix **W**_ee_. The connections from the excitatory to the inhibitory neuron populations are defined by **W**_ie_, and the reciprocal connections are given by **W**_ei_. All column-to-column connections, both within and across fields, are assumed to be excitatory. The inhibitory populations make only local, short-range connections within the cortical column. Lateral inhibition across columns within a field is mediated by the excitatory population of each column exciting the inhibitory populations of neighbouring columns. The state equations are:


(1) $$\tau_{\text{m}}\,\dot{\text{u}}\,\left(t\right)=-\text{u}\,\left(t\right)+{\text{W}}_{\text{ee}}\,\text{Q}\,\left(t\right)\cdot g\,\left[\text{u}\,\left(t\right)\right]-{\text{W}}_{\text{ei}}\,\text{Q}\,\left(t\right)\cdot g\,\left[u\,\left(t\right)\right]+i_{\text{aff}}\,\left(t\right),$$

(2) $$\tau_{\text{m}}\,\dot{\text{v}}\,\left(t\right)=-\text{v}\,\left(t\right)\,{\text{W}}_{\text{ie}}\,g\,\left[\text{u}\,\left(t\right)\right],$$

where ***u***(*t*) and ***v***(*t*) are vectors (224 × 1) of the state variables *u* and *v*, respectively, and τ_m_ = 30 ms is the membrane time constant. The term *i*_aff_(*t*) represents afferent sensory input. This input is tonotopically organised into 16 frequency channels (*c*_*f*_ = 1…16) which represent the activity of the inferior colliculus. This targets the MGN field through topographically organised connections so that each unit essentially represents a frequency channel. Because the various fields are topographically connected to each other, the cortical columns exhibit tonotopic organization in their responses, with the tuning curves becoming broader as one moves from MGN toward the parabelt. **Q** expresses STSD which drives adaptation. It is a diagonal 224 × 224 matrix where the diagonal elements are described by the 224-element vector **q**(*t*) of synaptic efficacies:


(3) $$\dot{\text{q}}\,\left(t\right)=-\frac{\text{q}\,\left(t\right)\,g\,\left[\text{u}\,\left(t\right)\right]}{\tau_{\text{o}}}+\frac{1-\text{q}\,\left(t\right)}{\tau_{\text{rec}}},$$

where the first r.h.s. term describes the fast onset of STSD and the second term encapsulates the slow recovery. Note that STSD is assumed to depend on the presynaptic firing rate only, and therefore all the connections originating from the same column are modulated by the same element of **q** (hence **q** is a 224-element vector). There are two time constants: τ_o_ is the onset time constant (100 ms in cortex, 20 ms in MGN), and τ_rec_ is the time constant of recovery. The recovery time constant was treated as a free variable, justified by N1 recovery being highly subject-specific (Lü et al., 1992; Ioannides et al., 2003). The respective values of τ_rec_ across Experiments 1–5 described below were: [1.2, 1.2, 1.2, 1.7, 1.4] s.

The MEG signal is to a large extent generated by dendritic current flowing in the apical dendrites of cortical pyramidal neurons (Hämäläinen et al., 1993). In the model, the MEG is approximated by spatially summing the excitatory input currents to the excitatory neuron populations, that is, the second term on the r.h.s. of Eq. 1 (for a detailed description, see Hajizadeh et al., 2019). In the summation, the contribution from each connection is weighted according to connection type, with the weights being [−2,1,1] for feedforward, feedback, and intra-field connections, respectively.

Five experiments were carried out with the following oddball stimulation:

**Experiment 1** – Standard stimuli (duration 50 ms, frequency channel *c*_*f*_ = 7) were presented with a stimulus onset interval (SOI) of 100 ms and omitted with 10% probability (parameters from Yabe et al., 1998). Each stimulus omission was treated as the deviant when calculating the ERF.

**Experiment 2** – The standard stimulation was a series of tones (duration 50 ms, SOI 500 ms) which alternated in *c*_*f*_ frequency between 6 and 9. Occasionally, the tone with *c*_*f*_ = 6 was repeated (*p* = 5%). Comparisons were made between the ERF response elicited by the *c*_*f*_ = 6 tone in these two cases.

**Experiment 3** – In the “global deviance” setup, two types of stimuli were used: a sequence of five identical tones (“xxxxX”; duration 50 ms, SOA 150 ms, *c*_*f*_ = 5) and a sequence “xxxxY” that was otherwise the same as xxxxX except that the fifth tone (*c*_*f*_ = 12) differed in frequency from the first four tones and was therefore a “local” deviant. These stimuli were presented in two conditions: one where xxxxY was the standard (*p* = 75%) and xxxxX was the “global” deviant (*p* = 15%), and one where these roles were reversed. In addition, the blocks contained occasional four-tone sequences (*p* = 10%). The sequences were separated by silent 850-ms periods. The parameters are from Wacongne et al. (2011).

**Experiment 4** – Standards (*c*_*f*_ = 9, *p* = 90%) and deviants (*c*_*f*_ = 10, *p* = 10%) were presented in the oddball paradigm (tone duration 50 ms, SOI = 500 ms). In a separate multi-standard control condition, the standards were randomly replaced with equiprobable tones of different frequencies (*c*_*f*_ = 4…13, *p* = 10% for each).

**Experiment 5** – Standards (*c*_*f*_ = 6, *p* = 80%) and deviants (*c*_*f*_ = 9, *p* = 20%) were presented as a series of anisochronous stimuli (duration 300 ms). The silent interval between consecutive tones varied randomly between 200 and 1,000 ms (flat distribution). The parameters are from Schwartze et al. (2011).

In all experiments, simulations comprised at least 400 presentations per condition. The responses to standards and deviants were averaged separately. The resulting ERFs were baseline-corrected (100 ms) and highpass filtered at 1 Hz.

## The Adaptation Model in Action: Simulation Results

**FIGURE 2:**
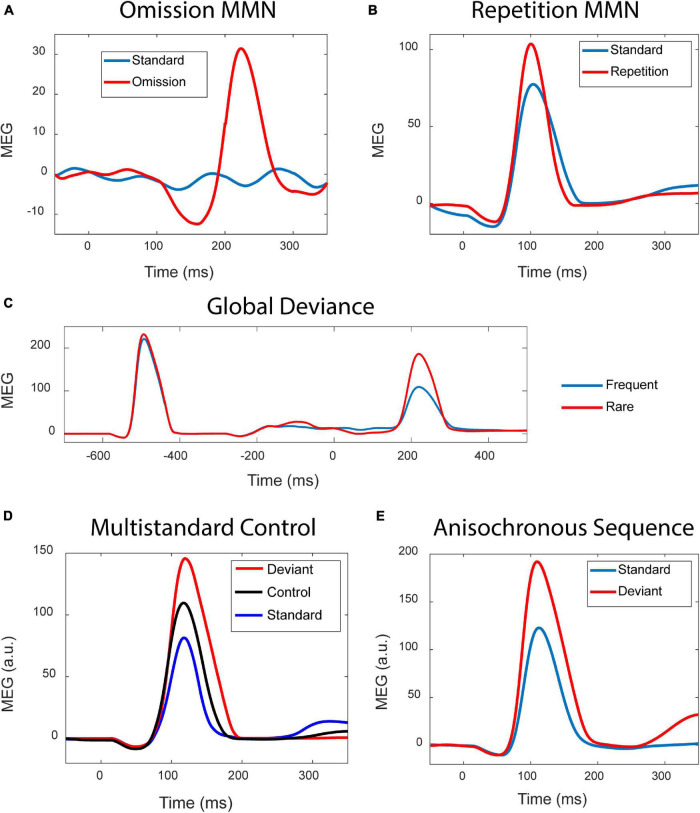
Simulation results. **(A)** Standard stimuli presented at a fast rate (blue curve) elicit no discernible response, whereas the occasional stimulus omission (red curve) results in a prominent MMN. **(B)** Occasionally repeating a tone (red) in a sequence of alternating tones (blue) results in an MMN. **(C)** The blue curve is the response to a sequence xxxxX of five tones presented as a global standard, and the red curve is the response elicited by the same xxxxX as an infrequent global deviant. When the sequence is a global deviant, the ending of the sequence elicits a much stronger response than when it is a global standard. Zero time indicates the onset of the fifth tone. **(D)** In the classic oddball paradigm, frequency deviants (red) elicit a stronger response than the standards (blue). The response to the deviants is also stronger than the response elicited by the same deviants when these are presented as part of a random sequence of tones, in the so-called multi-standard control condition (black). **(E)** Standards (blue) and deviants (red) were presented as a series of anisochronous stimuli where the SOI varied randomly.

Simulation results shown in Figure 2 demonstrate that the modern version of the adaptation model reproduces those types of MMN which previously have been taken as evidence against the adaptation hypothesis.

**Experiment 1** – The *omission MMN* is shown in Figure 2A. Due to the fast stimulus presentation rate, the standards (blue curve) produce no discernible responses. The occasional omission elicits a prominent response (red) which, apart from a late peak latency, resembles the observations of Yabe et al. (1998).

**Experiment 2** – Tones alternating in frequency served as the standard stimulation. Occasionally alteration was replaced by stimulus repetition. As shown in Figure 2B, this results in a *stimulus repetition MMN*, as was found in simulations of the PC model of Wacongne et al. (2012).

**Experiment 3** – Two types of sequences served as stimuli: five identical tones (xxxxX), and four identical tones followed by a “local” frequency deviant (xxxxY). Figure 2C shows the responses to the xxxxX sequence in two conditions: (1) It was the “global” standard stimulus, representing an expected repetition of the fifth tone. (2) It was the global deviant stimulus among xxxxY standards, therefore constituting an *unexpected stimulus repetition*. The global unexpectedness of the stimulus causes a late, “higher-order” MMN response, as observed by Wacongne et al. (2011).

**Experiment 4** – Figure 2D shows the results where the multi-standard control condition was utilised. The deviant in the oddball condition elicits a larger response (red) than it does in the control condition (black). This is surprising given that we are viewing the behaviour of the adaptation model.

**Experiment 5** – Figure 2E shows the results due to oddball stimulation. The frequency deviants (red) elicit stronger responses than the standards (blue). The twist here is that the presentation of the stimuli is anisochronous, with the stimulus onset intervals (SOIs) being random.

To summarise, the adaptation model produces a wide variety of MMNs which have been used as arguments against the adaptation hypothesis (Experiments 1–4). It is beyond the current scope to explore in detail what is generating the MMN in each experiment. As explained in May et al. (2015), SSA on the single-unit level is only part of the explanation, with tuning to stimulus features also playing a major role. Omission responses (Experiment 1) are to be expected as resonance effects, given that interacting excitatory and inhibitory neural populations are dynamically equivalent to driven oscillators with damping (May and Tiitinen, 2001; Hajizadeh et al., 2019, 2021). In addition, the omission response could be enhanced or even caused by high-pass filtering acting on the sudden, omission-related drop in the sustained activity which is elicited by fast-rate stimulation (May and Tiitinen, 2010). As for the multi-standard control results, these arise from the cortical columns being interconnected rather than acting as isolated frequency channels. Therefore, the response of each column depends not only on the stimulation rate (which would be required for the multi-standard control condition to be valid), but it is also modulated by lateral connections and the pattern of synaptic depression over the entire network, as established by the previous stimulation (May and Tiitinen, 2010). This means, for example, that columns that respond selectively to the standard-deviant combinations in the oddball condition respond less vigorously when this pattern is no longer dominant in the multi-standard condition, where the deviant is preceded by multiple different stimuli (May, 2017). This issue will be addressed in more detail in a separate paper.

## Adaptation, Predictive Coding, or a Bit of Both?

It is time to reconsider what we mean by the adaptation model of MMN. The traditional model posits that adaptation is merely the repetition suppression of individual isolated neural populations. This version is really just a straw man that we should abandon because the brain does not contain isolated populations. A modern, updated adaptation model can be encapsulated thus: There is no process, mechanism, cortical area, or set of pathways that is dedicated to MMN generation, functionally separate from the rest of auditory cortex. Instead, the physiological mechanism that causes repetition suppression of neural responses (e.g., of the N1), is the *same* as that which makes the MMN happen. The candidate for this mechanism is STSD, which on its own might seem low-level because it causes transient weakening of synaptic connections. However, the effect of these synaptic modulations on the system level is profound. This is because synaptic depression happens in the context of an intricately interconnected, hierarchically organised network containing both excitation and inhibition. The stimulation at any one time point leaves, via STSD, a slowly decaying, highly malleable imprint on the functional structure of auditory cortex, that is, on the multitude of synaptic strengths by which the auditory cortex neurons are connected to each other. This functional structure keeps evolving and, at any time point, represents a weighted integration of all the stimulation that has occurred in a time window stretching seconds into the past. Temporarily weakened excitatory connections thus contribute to an attenuated response if they belong to an excitatory feedback loop triggered by the incoming stimulation, but they can contribute to an enhanced response if the activated circuit drives inhibition. The response of a neuron in auditory cortex thus intertwines the effect of the stimulus with the effect of the stimulation history and in this way is specific to both stimulus and history. Therefore, repetition suppression is only one *of many* possible consequences of synaptic depression. These consequences show up as context sensitivity and, perhaps counterintuitively, as forward enhancement, depending on stimulation history (see May and Tiitinen, 2013; May et al., 2015). While STSD is a root cause of MMN, it plays a far wider role, enabling the gamut of other dependencies on stimulation history. Thus, there is nothing low-level about adaptation: while it is detected by using stimulus repetition – the simplest and the most boring of stimulation paradigms – it reflects a fundamental mechanism whereby the auditory cortex is able to keep track of the past in a way which informs the way it responds to the present.

The version of the adaptation model used here has a hierarchical structure in terms of the core, belt, and parabelt, and in the above simulations, the feedback connections are all excitatory. However, in contrast to the PC model, there is no requirement for the feedback to be exclusively inhibitory, and neither does it have to be exclusively excitatory; in either case (not shown here), the model of auditory cortex is able to generate MMN responses. The model suggests that the functional significance of the hierarchical structure of the auditory cortex lies in the way it modulates temporal binding. Namely, simulation results suggest that the time window over which combination sensitive responses occur increases as one moves up the core-belt-parabelt hierarchy (May and Tiitinen, 2013; May et al., 2015; Westö et al., 2016).

“Adaptation model” is somewhat of a misnomer because the object of modelling is *not* the MMN but, rather, the auditory cortex. Indeed, other modelling studies have similarly linked STSD in auditory cortex to SSA (Mill et al., 2011; Yarden and Nelken, 2017; Kudela et al., 2018) and to combination sensitivity (Lee and Buonomano, 2012; Goudar and Buonomano, 2015). Also, the current auditory cortex model is by no means complete. It lacks input from, for example, the inferior frontal cortex (IFC), which is known to contribute to the MMN response generated in auditory cortex (e.g., Rinne et al., 2005; Tse et al., 2018; Lui et al., 2021). The simulations can be taken as a demonstration that the “local” processing happening in auditory cortex is sufficient for the generation of MMN. There is no need to postulate a top-down generative model outside auditory cortex. However, it is still perfectly possible, even within the AM framework, that IFC and other areas have a modulatory role in shaping the MMN. Further, although mimicking the gross anatomy of auditory cortex, the model is an extremely simplified description, and it lacks, for example, long-term dynamics such as Hebbian learning. Nevertheless, it is noteworthy that such a simple model can mimic the behaviour of auditory cortex in so many ways and levels of observation.

Can PC claim similar success? Certainly, the results from Experiments 1–3 can be explained in terms of PC, as was done in the modelling work of Wacongne et al. (2012). However, explaining the omission MMN (Experiments 1 and 3) is not straightforward because there is no sensory signal for the prediction signal to suppress. Why, then, would there be an error signal? Wacongne and colleagues suggested that in this case, the MMN could reflect the activity of the prediction signal itself. This explanation is problematic because this signal should then be visible also when the prediction is successful, so that we would measure MMNs to standards too. Instead, as in the above simulation, the observed omission response tends to be more prominent than the responses to the standards (Yabe et al., 1997, 1998). Further, how does the generative model at the top of the hierarchy actually emerge? On this question, PC accounts are abstract and conceptual. For example, Wacongne and colleagues implemented the generative model as a set of delay lines which keep the stimulus-elicited signal in memory for precisely the right time so that the signal can then be recycled back as a top-down inhibitory prediction signal that coincides with the next stimulus. Noting that this delay-line scheme is unrealistic, the authors speculated that the generative model might in fact be due to parts of cortex acting like an echo state network. This is fair, and it will probably be a tremendous task to construct a mechanistic explanation of how the brain creates, on the fly, generative models to attempt to fit whatever the world is throwing at it. Even though the brain could be adept at doing this, given its pattern generating abilities, the generative model nevertheless currently plays the role of *deus ex machina* in PC theory. The existence of the generative model enthroned atop the hierarchy is assumed rather than explained. Research has concentrated on testing whether sensory responses are compatible with the PC view, and it remains unaccounted for how the past evidence is actually transformed into a projection of what the future most likely holds. One exception is the study by Friston and Kiebel (2009) where the generative model was a pair of Lorenz attractors offering an abundance of priors which could recover the hidden state of similar attractors driving the input. The input in this case was simulated bird song, which has a precise frequency and time structure. Thus, the requirement for the generative model was the ability to provide prediction signals with the right intricate timing. But how would such a precise system fare when the input arrives at random times, such as in Experiment 5 and in the MMN experiment of Schwartze et al. (2011)? This consideration is different from the one concerning precision weighting of the error signal. Rather, it concerns what form the actual generative model should take. By what mechanism would the generative model know when to employ temporal precision and when not to? Further, with repetitive stimulation, the N1 amplitude depends strongly on SOI: the rate of growth is strongest for shortest SOIs (<1 s) before levelling off with longer SOIs. This behaviour is easily replicated by the adaptation model (May et al., 2015). From a PC perspective, one would need to explain why the performance of the generative model deteriorates the fastest when modelling the regularity should be the easiest.

The physiological evidence for PC is mixed, and the theory has been criticized for being difficult to falsify (Walsh et al., 2020) – something the adaptation model also suffers from. There is thin evidence for the proposed separateness of neurons representing predictions and prediction errors (Heilbron and Chait, 2018) and it is unclear how PC might correlate with perception (Denham and Winkler, 2020). Therefore, while Bayesian inference seems to be a computational principle of the brain, the actual implementation of it is uncertain, with PC being one among many candidates (Rescorla, 2021). Perhaps a reformulated version of Bayesian inference incorporating the adaptation model might be worth considering. The pattern of STSD could be seen as a posterior model for sensory stimuli, though of course not a generative one. A separate version of the model will exist on each level of the hierarchy, updating itself based on local information. In this view, the MMN can still be seen as an error signal, but one perhaps targeting a generative model on the highest level of attention and action selection. It is possible that the brain uses local adaptation and PC in tandem but for different purposes: One the one hand, adaptation might be central to bottom-up change detection which drives involuntary attention shifts and is expressed in the MMN. On the other hand, PC might be the top-down mechanism which suppresses *task-irrelevant* signals in auditory cortex according to a generative model. This model would selectively describe those signals that need to be filtered out and this selection would be a function of the attentional set rather than just signal probability. Evidence for this kind of top-down, attention-related inhibition of sensory processing can be found in the visual system in the case of visual marking (Watson and Humphreys, 1997; Braithwaite and Humphreys, 2003, 2007), and it could be present in the auditory system also.

## Conclusion

It is too early to discard the adaptation model as an explanation of deviance detection as revealed in the MMN. Its modern version is able to reproduce a wide variety of MMN responses as well as intracortical results. PC as currently formulated provides a mostly conceptual explanation, and therefore it is difficult to contrast the relative successes of these models. Whilst the adaptation model is incomplete and it lacks the normative power and elegance of predictive coding, there are challenges ahead before the PC can match the adaptation model on a mechanistic level.

## Data Availability Statement

The original contributions presented in the study are included in the article. The simulation code is available at https://github.com/pjcmay/ACtx-Model. Further inquiries can be directed to the corresponding author.

## Author Contributions

PM programmed and ran the simulations, prepared the figures, made the tea, and wrote the manuscript.

## Conflict of Interest

The author declares that the research was conducted in the absence of any commercial or financial relationships that could be construed as a potential conflict of interest.

## Publisher’s Note

All claims expressed in this article are solely those of the authors and do not necessarily represent those of their affiliated organizations, or those of the publisher, the editors and the reviewers. Any product that may be evaluated in this article, or claim that may be made by its manufacturer, is not guaranteed or endorsed by the publisher.
